# New Medical Journals

**Published:** 1861-03

**Authors:** 


NEW MEDICAL JOURNALS.—The *Baltimore Journal of Medicine* made its appearance on the first of January, and will be issued bi-monthly, under the editorial management of Dr. Edward Warren, Professor of Materia Medica and Therapeutics in the University of Maryland. From the well-known ability of its editor, we bespeak for it a hearty welcome.

We have received the first two numbers of the *Berkshire Medical Journal*, a monthly issue of forty-eight pages, edited by Dr. Wm. Henry Thayer and Dr. R. Cresson

Stiles, Professors in the Berkshire Medical College. We take pleasure in placing it on our exchange list.

The *Medical Journal of North Carolina*, published under the auspices of the State Medical Society, has been entrusted to the editorial management of Dr. Charles E. Johnson and Dr. S. S. Satchwell.

The *Archives of Medicine*, edited by Dr. Lionel S. Beale, appears to be gaining favor with the profession, both at home and abroad. Part VI., which has just reached us, maintains the well-earned reputation of its predecessors, and contains, among other papers, a chapter in clinical medicine, by Dr. W. Ogle; cases of trephining in syphilitic diseases of the skull, by Mr. Henry Lee; a paper by Dr. John Ogle, on the so-called "false membranes" in connection with the coverings of the brain; a simple and accurate method of recording physical signs by Dr. Beale; and a good exposition of cellular pathology, by Dr. Duffin.

